# An in-vivo comparison of stimulated-echo and motion compensated spin-echo sequences for 3 T diffusion tensor cardiovascular magnetic resonance at multiple cardiac phases

**DOI:** 10.1186/s12968-017-0425-8

**Published:** 2018-01-03

**Authors:** Andrew D. Scott, Sonia Nielles-Vallespin, Pedro F. Ferreira, Zohya Khalique, Peter D. Gatehouse, Philip Kilner, Dudley J. Pennell, David N. Firmin

**Affiliations:** 1 Cardiovascular Magnetic Resonance Unit, Royal Brompton and Harefield NHS Foundation Trust and Imperial College London, Sydney Street, London, UK; 2 National Heart and Lung Institute, Imperial College London, Sydney Street, London, UK; 3 National Heart Lung and Blood Institute, National Institutes of Health, Bethesda, MD USA

**Keywords:** Diffusion, Diffusion tensor imaging, Cardiac, Heart, STEAM, Spin-echo, Motion compensation

## Abstract

**Background:**

Stimulated-echo (STEAM) and, more recently, motion-compensated spin-echo (M2-SE) techniques have been used for in-vivo diffusion tensor cardiovascular magnetic resonance (DT-CMR) assessment of cardiac microstructure. The two techniques differ in the length scales of diffusion interrogated, their signal-to-noise ratio efficiency and sensitivity to both motion and strain. Previous comparisons of the techniques have used high performance gradients at 1.5 T in a single cardiac phase. However, recent work using STEAM has demonstrated novel findings of microscopic dysfunction in cardiomyopathy patients, when DT-CMR was performed at multiple cardiac phases. We compare STEAM and M2-SE using a clinical 3 T scanner in three potentially clinically interesting cardiac phases.

**Methods:**

Breath hold mid-ventricular short-axis DT-CMR was performed in 15 subjects using M2-SE and STEAM at end-systole, systolic sweet-spot and diastasis. Success was defined by ≥50% of the myocardium demonstrating normal helix angles. From successful acquisitions DT-CMR results relating to tensor orientation, size and shape were compared between sequences and cardiac phases using non-parametric statistics. Strain information was obtained using cine spiral displacement encoding with stimulated echoes for comparison with DT-CMR results.

**Results:**

Acquisitions were successful in 98% of STEAM and 76% of M2-SE cases and visual helix angle (HA) map scores were higher for STEAM at the sweet-spot and diastasis. There were significant differences between sequences (*p* < 0.05) in mean diffusivity (MD), fractional anisotropy (FA), tensor mode, transmural HA gradient and absolute second eigenvector angle (E2A). Differences in E2A between systole and diastole correlated with peak radial strain for both sequences (*p* ≤ 0.01).

**Conclusion:**

M2-SE and STEAM can be performed equally well at peak systole at 3 T using standard gradients, but at the sweet-spot and diastole STEAM is more reliable and image quality scores are higher. Differences in DT-CMR results are potentially due to differences in motion sensitivity and the longer diffusion time of STEAM, although the latter appears to be the dominant factor. The benefits of both sequences should be considered when planning future studies and sequence and cardiac phase specific normal ranges should be used for comparison.

**Electronic supplementary material:**

The online version of this article (10.1186/s12968-017-0425-8) contains supplementary material, which is available to authorized users.

## Background

There is a growing interest in diffusion tensor cardiovascular magnetic resonance (DT-CMR) due to its unique ability to noninvasively interrogate the myocardial microstructure, and recent publications have demonstrated potential clinical applications [1–7]. However, the dynamic nature of the heart makes it a challenging target for DT-CMR. Initial in-vivo DT-CMR studies used a stimulated echo acquisition mode (STEAM) based echo-planar imaging (EPI) technique, with diffusion encoding gradients at identical trigger times in two successive cardiac cycles [8, 9]. Diffusion is encoded over one complete cardiac cycle (the diffusion time, Δ) and relatively large diffusion weightings (b-values) can be achieved with relatively small gradient pulses. This allows STEAM DT-CMR to be performed at time points throughout the cardiac cycle [6, 10], which has been used to demonstrate impairment of the rotation of the aggregates of cardiomyocytes known as sheetlets in hypertrophic and dilated cardiomyopathy patients [6, 7, 11]. A recent validation study [6] demonstrated excellent similarity between angular DT-CMR measures obtained in hearts imaged while beating and arrested in-vivo, ex-vivo and histologically in relaxed and contracted states. However, differences in myocardial position and conformation between the diffusion encoding gradients result in changes in image phase and signal intensity. This makes studies difficult during arrhythmia or during free-breathing [9]. The measured diffusion is also affected by the strain history of the myocardium between the encoding gradients [12] although the size of this effect is under debate [1, 6, 13].

To deal with the effects of strain on the diffusion tensor a correction model has been used with input from 3D strain data [12]. Alternatively, using the same model, times in the cardiac cycle where strain has no effect (so called strain “sweet-spots”) on measured diffusion can be identified from strain data [14]. These techniques also require a measurement of 3D strain throughout the cardiac cycle.

Another approach is to use a spin-echo (SE) sequence similar to neurological diffusion tensor techniques [15, 16]. The duration of the diffusion gradients when using SE sequences is usually too long to identify a matching quiescent cardiac phase, but motion compensation may be incorporated into the diffusion encoding gradients [17]. Recently, velocity and acceleration compensated (M2) [18, 19] gradients have been used to perform SE DT-CMR in vivo [20, 21]. A comparison between STEAM and M2-SE techniques [21] described improved SNR efficiency and a more linear transmural distribution of helix angles using M2-SE, as well as differences in fractional anisotropy (FA) and mean diffusivity (MD) between sequences. The short diffusion time of M2-SE techniques means that the technique does not require two cardiac cycles per image, strain effects are minimised and the 50% signal loss inherent to stimulated echoes is avoided. The long diffusion encoding gradient pulses for M2-SE avoid the T1 signal loss during the time between the second and third radiofrequency (RF) pulses (the mixing time) but result in long echo times (TE) and consequential signal loss. However, previously published in-vivo human demonstrations of M2-SE DT-CMR have predominantly used high performance gradient systems at 1.5 T [20, 21].

In this work we implement an M2-SE DT-CMR sequence on a 3 T clinical CMR scanner with standard gradients and directly compare to the STEAM sequence using matched protocols at three cardiac phases. We also consider the contribution of strain to the differences in DT-CMR results between the cardiac phases and sequences.

## Methods

Fifteen healthy subjects (9 male, median age 24, range 20–36) were recruited with consent according to ethical approval. Images were acquired using a Siemens 3 T scanner (Skyra, Siemens Healthineers, Erlangen, Germany) with standard gradients (43mT⋅m^−1^ and 180T⋅[m⋅s]^−1^ per axis) using anterior (18-elements) and posterior (8–12 elements) RF coils. Balanced steady-state free-precession cine imaging was used to identify a mid-ventricular short-axis plane and the end-systolic and end-diastolic periods for subsequent acquisitions.

### DT-CMR sequences

**Fig. 1:**
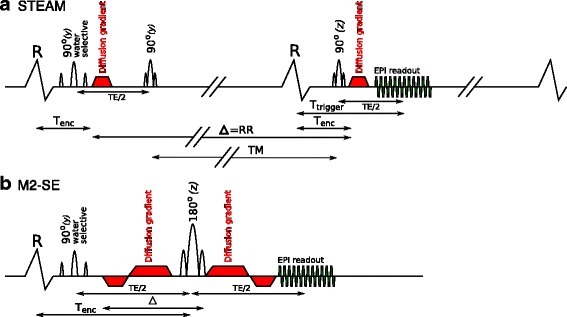
Comparison of sequence schematics. The STEAM sequence (**a**) runs over 2 cardiac cycles and the M2-SE (**b**) is triggered to every R-wave (labelled R here). The letters above the radiofrequency (RF) pulses indicate the axis that the corresponding slice selective gradient is played out. T_enc_ – time from R-wave to effective time of diffusion encoding, TM – mixing time, RR – RR-interval, T_trigger_ – time from R-wave to central k-space line, Δ – the diffusion time of the sequence

The STEAM EPI DT-CMR sequence was described in previous work [9] (Fig. 1a). A breath-hold SE EPI DT-CMR sequence was implemented with first and second order motion compensated diffusion encoding gradients (M2-SE) to minimise signal loss artefacts caused by constant velocity and acceleration during the gradients (Fig. 1b). The gradient design was based on that described by Welsh et al. [18] and Stoeck et al. [20].

To facilitate comparisons, imaging parameters were matched between sequences. For both sequences, EPI readout duration was reduced by a “zone-selected” technique [22]. A sensitivity encoding (SENSE) [23] acceleration factor of 2 was used to further reduce the EPI readout length. No partial Fourier sampling was used. Identical EPI readouts were used for both sequences, with a 13 ms duration, 2442 Hz/pixel bandwidth and 0.51 ms echo spacing. Spatial resolution was 2.8 × 2.8mm^2^, 1.4 × 1.4mm^2^ via zero-filled reconstruction and 8 mm slice thickness. Investigation of the sequence used in a previous comparison [24] demonstrated a mismatch in the effective slice thickness between the techniques. For this comparison, sinc shaped pulses were used for the final RF pulse in both sequences and the full-width half-maximum of the slice profile was adjusted to 8 mm. The M2-SE sequence acquires one spin-echo every cardiac cycle (RR-interval), whereas STEAM acquires a stimulated echo every other cardiac cycle. In each breath-hold the first spin or stimulated echo is used for EPI phase correction lines and the second for parallel imaging references data. Then, the “b0” image is acquired (where diffusion encoding is replaced with spoiler gradients; b = 34s⋅mm^−2^ for STEAM and b = 30s⋅mm^−2^ for M2-SE), followed by each of the 6 diffusion encoding directions used (7 or 14 RR intervals for M2-SE or STEAM respectively). In order to produce similar breath-hold durations, two averages per breath-hold were acquired for M2-SE (total 16RR-intervals vs. 18RR-intervals for STEAM). Based on previous M2-SE studies b_main_ = 450s⋅mm^−2^ was used for both sequences. The total durations were 2.4 ms for each of the STEAM diffusion encoding gradients and 9.1 and 17.5 ms for each of the short and long M2-SE diffusion encoding gradients. Rather than using “b0” images in the tensor calculation, additional data was acquired for use as the reference with b_ref_ = 150s⋅mm^−2^ in the same 6 directions as the b_main_ data [20, 25]. For STEAM 8 averages and for M2-SE 16 averages (8 breath holds) were used for b_main_ and 1 (STEAM) or 2 (M2-SE) averages (1 breath-hold) were used for b_ref_. Acquisition was triggered to alternate R-waves (TR = 2 RR-intervals) for STEAM and every R-wave for M2-SE (TR = 1 RR-interval). A 1–2-1 binomial water excitation RF pulse was used for the initial excitation in both sequences. Flip angles were 90° in the STEAM sequence and 90° – 180° in the M2-SE sequence. TE was 25 ms for STEAM and 76 ms for M2-SE.

### Phantom signal to noise ratio measurements

Signal-to-noise ratio (SNR) was compared between the sequences using a cylindrical agar phantom (13 cm diameter, 40g⋅L^−1^ agar in tap water). Transverse images were acquired (50 averages) with the phantom axis aligned with the magnetic field. Imaging parameters are as described above with a simulated RR-interval of 1000 ms. The “b0” images (b_ref_ = 34s⋅mm^−2^, STEAM and b_ref_ = 30s⋅mm^−2^, M2-SE) were compared. Pixelwise SNR per image was calculated as the ratio of the mean to standard deviation of the signal in each pixel over time [26] and the mean value was taken from a region of interest (ROI) drawn in the centre of the phantom. Adapting the theory described by von Deuster et al. [21] (who compared SNR efficiency), the ratio of the mean SNR per image in the phantom was compared to the theoretical value calculated via:

1 $$ \frac{SNR_{SE}}{SNR_{STEAM}}=\frac{\left(1-{e}^{\frac{-{Trecov}_{SE}}{T1}}\right)\kern0.5em \cdotp {e}^{\frac{-{TE}_{SE}}{T2}}\kern0.5em \cdotp {e}^{-{b}_{SE}\cdotp D}}{\frac{1}{2}\left(1-{e}^{\frac{-{Trecov}_{STEAM}}{T1}}\right)\kern0.5em \cdotp {e}^{\frac{-{TE}_{STEAM}}{T2}}\kern0.5em \cdotp {e}^{-{b}_{STEAM}\cdotp D}\kern0.5em \cdotp {e}^{\frac{- TM}{T1}}} $$

where *Trecov*_*SE*_ and *Trecov*_*STEAM*_ are the inter-shot longitudinal recovery times for the M2-SE and STEAM sequences respectively (1RR interval in both cases), TE_SE_ and TE_STEAM_ are TE for each of the sequences, D is the diffusivity of the agar phantom, *b*_*SE*_ and *b*_*STEAM*_ are the b-values for each of the sequences and TM is the mixing time of the STEAM sequence (1 RR-interval). A flip angle of 90° was assumed for each of the STEAM RF pulses and 90° – 180° for the pulses in the M2-SE sequence.

### In-vivo imaging

DT-CMR was performed at systole, diastole and the systolic sweet-spot using both STEAM and M2-SE. For the M2-SE sequence, systolic acquisitions were timed to acquire the centre of k-space at end systole and diastolic acquisitions were timed to place diffusion encoding gradients and image data acquisition during the most stationary period of diastole (assessed visually on cine images). Where images from the first M2-SE breath-hold were poor quality in systole or diastole, the acquisition was optimised by repetition at trigger delays ±20 ms. STEAM data for the systolic or diastolic timing were acquired immediately after the corresponding M2-SE acquisition and trigger times were set to match the timing of the centre of the diffusion encoding gradients between sequences. Based on our previous work [27] and the mean sweet-spot timing used in [21] of 142 ms we used a fixed time of 150 ms from the R-wave to the centre of the diffusion encoding for sweet-spot imaging. While the M2-SE acquisition timing was optimised and data was acquired before the STEAM data at each cardiac phase, the order in which the cardiac phases were acquired was randomised between subjects.

### Strain

Breath-hold spiral cine displacement encoding with stimulated echoes (DENSE) [28, 29] was performed with a novel sequence accelerated by using a reduced field of view in two dimensions [30]. 2D acquisitions with 2-direction encoding were performed in the same plane as the DT-CMR acquisitions at a 3.3 × 3.3mm^2^ spatial resolution, 30 ms temporal resolution, 8 mm slice thickness, 224x224mm^2^ field of view and 14 RR-interval breath holds. A variable flip angle with a maximum of 20^o^ was used, chemical shift selective fat saturation, TE = 1 ms, TR = 15 ms, 2 spiral interleaves per temporal frame, 6 ms per spiral and 4 interleaves per image. Displacement encoding frequency was 0.06cycles⋅mm^−1^ and artefacts from unwanted echo pathways were minimised using CSPAMM-like encoding and through-plane spoiling (0.08cycles⋅mm^−1^) [31].

### Processing

DT-CMR data was processed using in-house MATLAB (Mathworks, Natick, Massachusetts, USA) software as described in previous work [1, 25, 32, 33]. The b_ref_ (150s⋅mm^−2^) and b_main_ (450s⋅mm^−2^) images were used to calculate the diffusion tensor. Frames corrupted by motion were discarded after visual assessment and the images were registered using a rigid translation. A variable amount of myocardial blood signal was present in the M2-SE images. Pixels containing blood with a very high signal intensity were nulled based on their intensity prior to the image registration step and the original signal intensities were returned after registration. A diffusion tensor was calculated at every pixel from the registered magnitude images using a linear least squares inversion. Each image was included separately within the matrix inversion without averaging and for the STEAM sequence the b-values were corrected for the RR-interval on a beat-to-beat basis.

Pixel-wise maps of helical angle (HA), absolute value of the second eigenvector angle (E2A) [1], fractional anisotropy (FA), tensor mode (mode) [34], mean diffusivity (MD) and each of the three eigenvalues (E1, E2, E3) were calculated. SNR per image was calculated in an ROI in the septal mesocardium (away from blood signal and artefacts) in the “b0” images (b = 34s⋅mm^−2^ for STEAM [9 images] and b = 30s⋅mm^−2^ for M2-SE [18 images]) without averaging using the repeated measurement technique [9, 26]. To facilitate quantitative comparisons of HA, wall thickness normalised helical angle gradient (HAG) in units of ^o^/% was calculated from epi- to endocardium radial profiles [35, 36]. For comparative analysis, mean left ventricular (LV) values for all parameters except E2A were calculated after removing papillary muscles and the right ventricular portion of the septum. Median LV E2A values were used because E2A is not expected to be normally distributed.

DT-CMR data quality was assessed by scoring the HA maps on the assumption of a circumferential linear variation of HA from epi- to endocardium. A score of 3 was given to HA maps with visually >95% normal transmural HA variation, 2 for >75%, 1 for >50% and 0 for <50%. Acquisitions with scores of 0 were considered a failure and excluded from further analysis. Scoring was performed separately in a randomised order by two blinded observers (4 and 2 years of DT-CMR experience) and conflicts were resolved by consensus. Based on the assumption of a constant transverse angle within each slice and a linear transmural HA profile, the standard deviation of the transverse angle [21] (TA std) and both the Pearson R^2^ and root mean squared error of the linear regression of the transmural HA profiles (HA R^2^ and HA RMSE) were used as quantitative DT-CMR quality measures.

Global peak radial and circumferential LV strains were calculated from the cine spiral DENSE data using DENSE Analysis software [37, 38]. We compared peak strains with the differences in diffusion derived parameters (E2A, HAG, MD, FA and mode) between systole and diastole, and between the sequences at each cardiac phase.

### Statistical analysis

All statistics were calculated in MATLAB (Mathworks). Global LV DT-CMR results were compared using a Wilcoxon test between sequences at systole, sweet-spot and diastole. Results were compared between cardiac phases using Friedman test with Bonferroni corrected Wilcoxon pairwise comparisons. Differences between DT-CMR results in systole and diastole and between techniques were correlated with peak radial and circumferential strains (Pearson correlation coefficient). *P* < 0.05 was considered significant.

## Results

### Phantom study

The phantom was found to have a T1 = 1090 ms (modified Look-Locker imaging, 5(3)3 [39, 40]) and T2 = 51 ms (repeated spin-echo acquisitions with increasing TE) and a mean diffusivity of 1.2 × 10^−3^mm^2^⋅s^−1^ (product Stejskal-Tanner spin-echo EPI sequence). The resulting theoretical SNR ratio (M2-SE/STEAM) was 1.85 and the measured value (mean ± standard deviation within an ROI) was 1.75 ± 1.14.

### In-vivo imaging

**Fig. 2:**
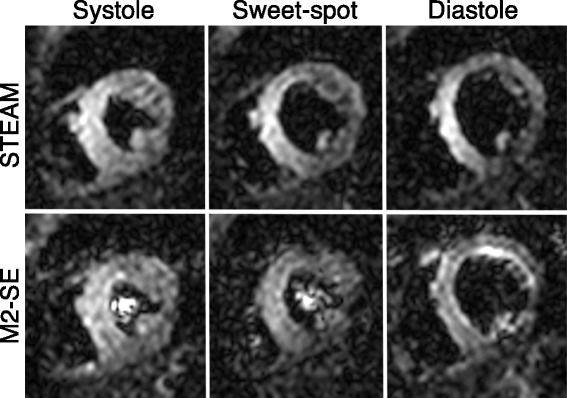
Typical raw image quality. Images are shown without averaging at b = 450smm^−2^ obtained from both of the sequences at each of the three cardiac phases in the cardiac cycle tested. Each of the images was acquired with diffusion encoding in the same direction and the images are windowed based on equalising the mean image intensity in an ROI drawn in septum. The bright left ventricular (LV) blood pool is evident in the M2-SE images due to the incomplete nulling of the blood signal. There is a higher signal intensity in the septal wall than in the lateral wall in images from both sequence. This is likely to be the result of spatial variation in the in-plane excitation profile (phase encode direction is horizontal in the image plane shown), variations in parallel imaging g-factor and uncorrected components of spatial variation in coil sensitivity

**Fig. 3:**
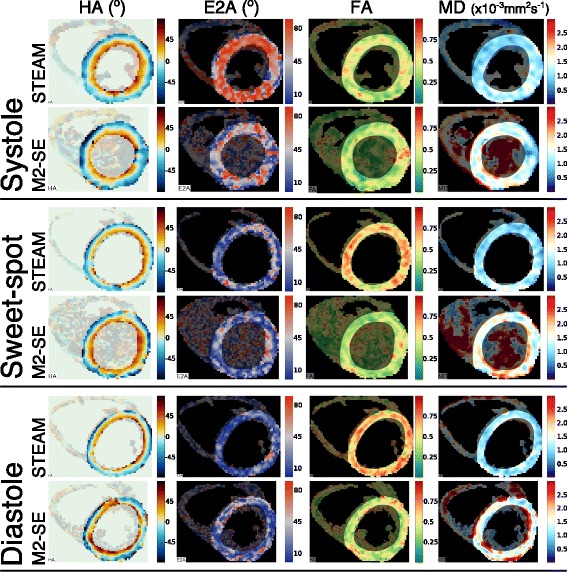
Example of successful DT-CMR maps. Results are shown from both sequences at all three cardiac phases from a subject where image quality was good in all acquisitions. The parameter maps are masked to indicate the region of the LV used in the analysis

**Fig. 4:**
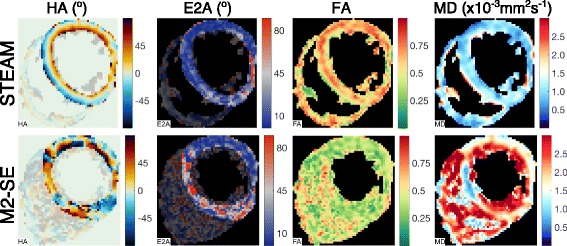
Example of unsuccessful M2-SE DT-CMR parameter maps. Maps are shown from a subject where M2-SE imaging was unsuccessful in diastole (scored 0), but diastolic STEAM imaging in the same subject scored 3. The raw images for these two datasets are provided in Supplementary Fig. S1 and S2 for the STEAM and M2-SE acquisitions respectively

**Fig. 5:**
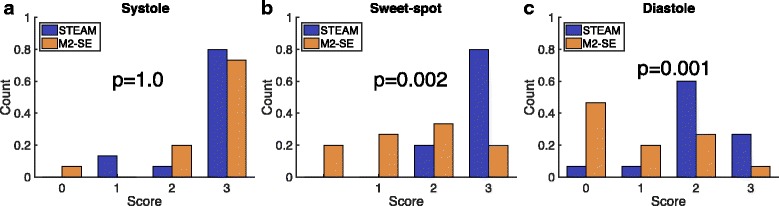
Image quality score results. The results of visual image quality scores based on helix angle (HA) maps shown as histograms for systolic (**a**), sweet-spot (**b**) and diastolic (**c**) data normalised to the number of acquisitions. Each HA map was visually scored from 0 (<50% of the myocardium demonstrating a normal transmural HA progression) to 3 (>95% normal HA progression). Statistical comparisons between the image scores at each cardiac phase were performed using the Wilcoxon signed-rank test and the corresponding *p*-value is shown on each plot

DT-CMR studies were of a sufficient quality for further analysis in 44/45 STEAM studies (1 diastolic acquisition scored 0) and in 34/45 M2-SE studies (14/15 systolic, 12/15 sweet-spot, and 8/15 diastolic). Figure 2 shows the typical image quality for raw unaveraged images. Examples of good quality DT-CMR parameter maps are shown in Fig. 3. For contrast, a diastolic example where STEAM was successful (scored 3), but M2-SE was of poor quality (scored 0) is shown in Fig. 4 (with corresponding raw images shown in Additional file 1: Figure S1 and S2). The median image quality at each cardiac phase was (STEAM/M2-SE): 3/3 for systole, 3/2 for sweet-spot, 2/1 for diastole. Histograms of the image quality scores are shown in Fig. 5 and scores were significantly different between the sequences at the sweet-spot and in diastole (*p* < 0.01). Mean ± standard deviation RR-interval was 970 ± 140 ms. There was a significant correlation (Spearman) between the image quality score and the RR-interval for the STEAM data at the sweet spot (Rho = 0.58, *p* = 0.02) and for M2-SE data in diastole (Rho = 0.73, *p* = 0.002), see Additional file 1: Figure S3. Correlations between mean RR-interval and image quality score were not significant for other combinations of sequence and cardiac phase or between image score and the intra-subject RR-interval variability (standard deviation).

**Fig. 6:**
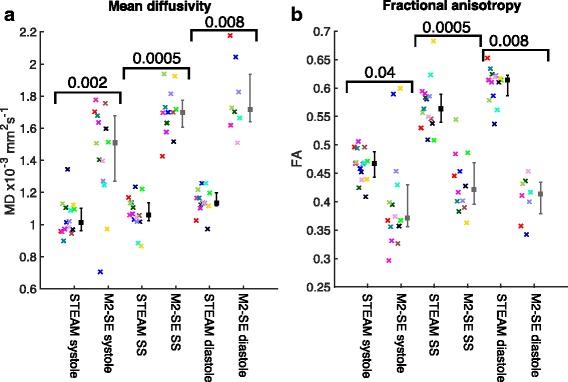
Mean diffusivity and fractional anisotropy results. A comparison of the mean LV MD (**a**) and FA (**b**) obtained from both sequences using the DT-CMR data at all three cardiac phases. The points are colour coded by subject, median and interquartile ranges are shown in black (STEAM) and grey (M2-SE) with the p-value obtained from a Wilcoxon signed rank test comparing the sequences at each cardiac phase shown above the data

**Fig. 7:**
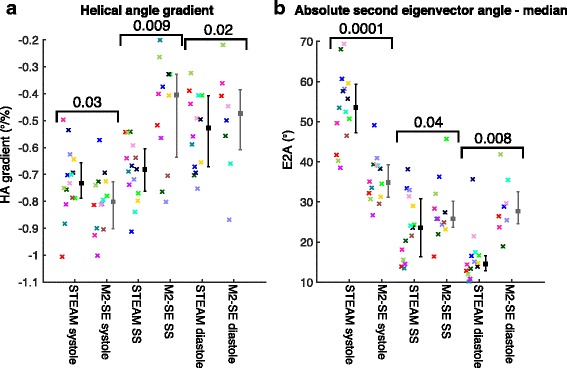
Helix angle gradient and E2A results. A comparison of the mean LV helical angle gradient (HAG) (**a**) and second eigenvector angle (E2A) (**b**) obtained from both sequences using the DT-CMR data at all three cardiac phases in the cardiac cycle. The points are colour coded by subject, median and interquartile ranges are shown in black (STEAM) and grey (M2-SE) with the p-value obtained from a Wilcoxon signed rank test comparing the sequences at each cardiac phase shown above the data

Global LV values of MD and FA are shown in fig. 6 and both HAG and E2A are plotted in fig. 7. Global tensor mode is plotted in Additional file 1: Figure S4; TA std., HA R^2^ and HA RMSE in SAdditional file 1: Figure S5; and SNR per image in a mesocardial septal ROI and mean signal in the same ROI are plotted in Supporting Fig. S6. The statistics shown in the figures provide a comparison between sequences at each cardiac phase while Supporting Table S1 compares the cardiac phases for each sequence. Additional file 1: Table S1 includes E2A measures obtained using both the median and mean over the LV to allow for comparison with previous work [1, 30, 31]. There are significant differences (*p* < 0.05) between the two sequences at all three cardiac phases for MD, FA, tensor mode, HAG and E2A. The non-subjective data quality measures were suggestive of better quality STEAM data at the sweet-spot (TA std. and HA RMSE significantly lower and HA R^2^ significantly higher, p < 0.05) and TA std. was suggestive of better quality diastolic STEAM data (p < 0.05). SNR per image was significantly higher at all three cardiac phases for STEAM (*p* < 0.01), but there were no significant differences between sequences in the mean signal. For the STEAM acquisitions there were significant differences (p < 0.05 or *p* < 0.017 after Bonferroni correction) between all three cardiac phases for E2A and E1; between systole and both sweet-spot and diastole for FA and E3; between diastole and both sweet-spot and systole for HA R^2^ and HAG; between systole and diastole for MD; and between systole and both sweet-spot and diastole for TA std. For the M2-SE acquisitions there were significant differences between cardiac phases only for SNR per image.

**Fig. 8:**
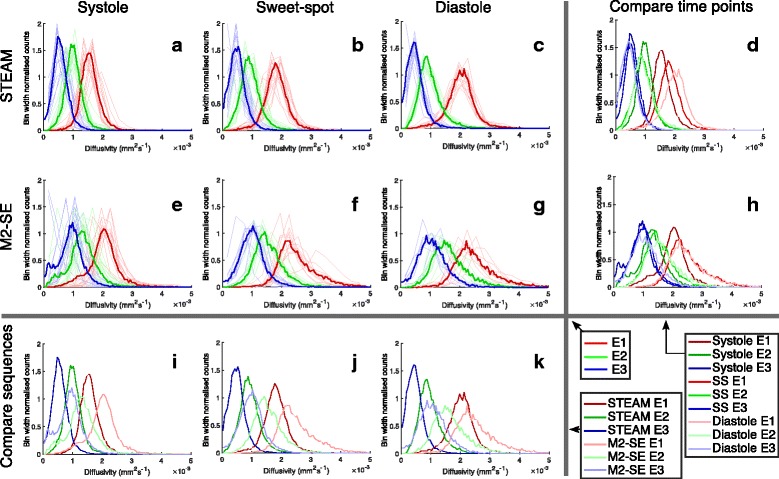
Eigenvalue histograms. Histograms of the eigenvalues obtained at each cardiac phase using both sequences. **a**–**c** and **e**–**g** show the histograms for the individual subjects (dashed lines) at each cardiac phase for STEAM and M2-SE respectively. The mean values are shown as solid lines. **d** and **h** compare the average histograms acquired at each cardiac phase for STEAM and M2-SE respectively. **i**, **j** and **k** compare the average histograms between the two sequences acquired at each cardiac phase. Each histogram was normalised to the number of samples (pixels) and then to the bin width used (0.3 × 10^−3^mm^2^⋅s^−1^ for individual subjects and 0.05 × 10^−3^mm^2^⋅s^−1^ for averaged histograms), giving y-axis units of 1/(10^−3^mm^2^⋅s^−1^)

The increased MD and reduced FA using the M2-SE sequence is reflected in the larger eigenvalues with smaller relative differences between them when compared to the STEAM results. In general, the eigenvalue histograms in Fig. 8 show less variation between subjects using the STEAM sequence than M2-SE and the average histograms for M2-SE have a broader peak as a result. The upper tail of the primary eigenvalue histogram for the STEAM sequence extends to around 3 × 10^−3^mm^2^⋅s^−1^, which is the diffusivity of free water at 37 °C [41]. In contrast, the tail of the primary eigenvector distribution extends to around 5 × 10^−3^mm^2^⋅s^−1^ for the M2-SE sequence.

**Fig. 9:**
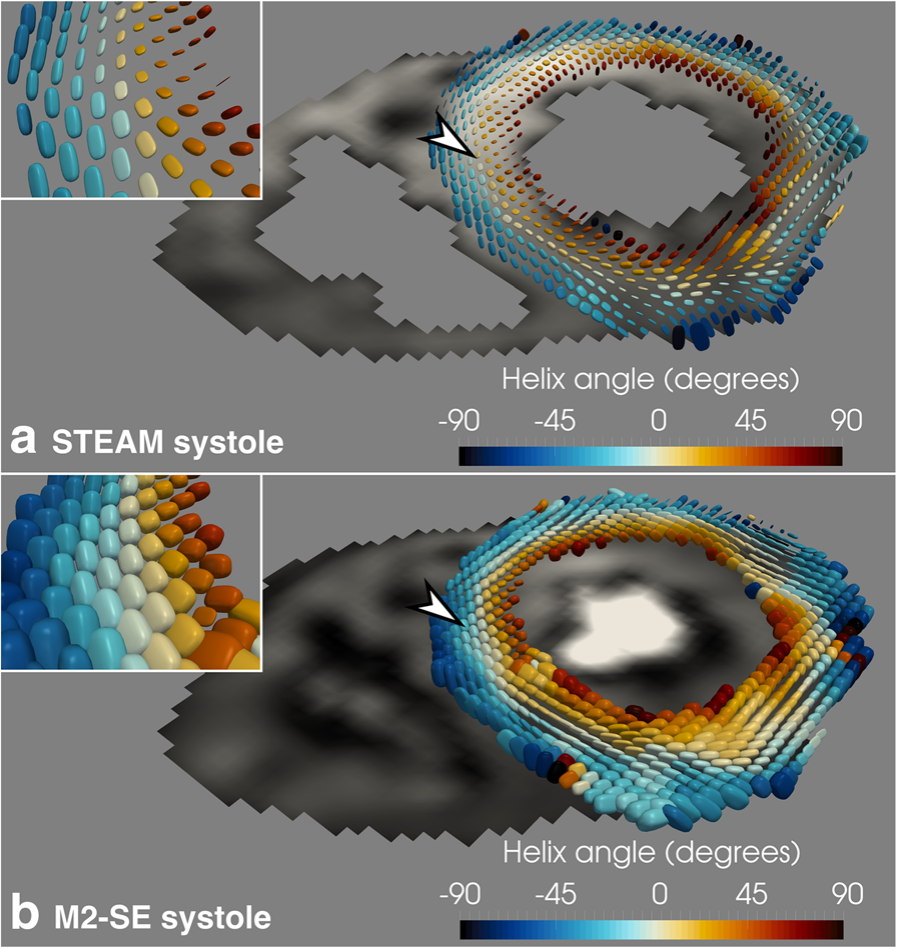
Superquadric glyph comparison. Example superquadric glyphs from both the STEAM (**a**) and M2-SE (**b**) sequences in systole. The elevated MD and reduced FA using M2-SE is evident in the zoomed inset regions as more cube like glyphs. The glyphs in the mid-wall of (**b**) are more perpendicular to the imaging plane due to the reduced E2A when using M2-SE. Arrow heads highlight the approximate centres of the inset regions on the main views

The tensor differences between the two sequences are also reflected in the systolic superquadric glyph representation of the diffusion tensor shown in Fig. 9.

**Fig. 10:**
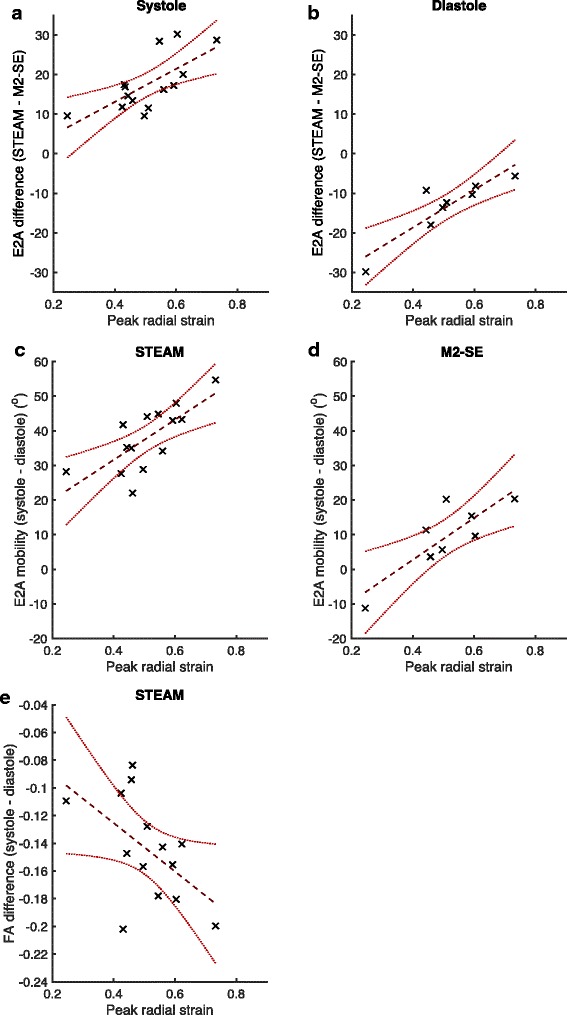
Significant correlations with strain. Scatter plots and linear regressions for all significant (*p* < 0.05) correlations between differences in DT-CMR parameters (between STEAM and M2-SE [**a**, **b**] or between systole and diastole [**c**–**e**]) and peak radial strain obtained from the 2D DENSE data. Linear regressions are shown as dashed lines and the 95% confidence intervals (functional non-simultaneous bounds) for the regression are shown in red dotted lines. Details of the fit parameters are given in Supporting Table S2

The median [interquartile range] peak strains were 0.50 [0.15] and −0.177 [0.016] in the radial and circumferential directions respectively. Additional file 1: Table S2 lists all the correlations with *p* < 0.05 obtained when comparing the differences in DT-CMR results (systole – diastole and STEAM – M2-SE) with peak radial and circumferential strains and the corresponding plots are shown in Fig. 10. No significant correlations were found when comparing the sequence or cardiac phase differences with peak circumferential strain. Using STEAM, the difference in both FA and E2A between systole and diastole significantly correlates with peak radial strain (*p* = 0.05 and *p* = 0.003 respectively). Using M2-SE, the significant correlation between E2A difference and peak radial strain is still present (*p* = 0.01). The E2A difference between the two sequences also correlates with peak radial strain in both systole and diastole (*p* < 0.01).

## Discussion

In this work we have implemented a 1st and 2nd order motion compensated (M2) SE DT-CMR sequence and compared results to those obtained from a monopolar STEAM sequence at 3 points in the cardiac cycle. To the best of our knowledge, this is the first published work to compare these sequences at multiple points in the cardiac cycle. While standard performance gradients have been used for velocity and acceleration compensated (M2) diffusion prepared techniques at 3 T [5, 19] and M2-SE has been demonstrated with a range of gradients strengths at 1.5 T during systolic contraction [20, 21, 42], this is the first study to demonstrate M2-SE with standard performance gradients at 3 T. While we were able to perform both sequences at all cardiac phases in most subjects, there were clear differences in reliability, image quality and the DT-CMR results.

For the healthy cohort in this work, the sequences performed similarly in systole, but STEAM was more reliable and image quality scores and quantitative measures were suggestive of better quality data in the sweet spot and diastole. A substantial number of M2-SE acquisitions were deemed to have failed (7/15 in diastole). In contrast, STEAM was successful in all but 1 acquisition and the visual image quality scores were significantly higher for the STEAM sequence at the sweet-spot (*p* = 0.002) and diastole (*p* = 0.001).

There are differences between the values of DT-CMR results the two sequences provide. Significant differences were present in FA, MD, tensor mode, E2A and HAG at all three cardiac phases (all *p* < 0.05). This is to be expected given the differences between the two approaches. The longer TE of M2-SE (76 ms vs. 25 ms STEAM) results in greater T2 weighting of the images. The reference and diffusion-weighted images used to calculate the diffusion tensor use the same TE and, therefore cancel the effects of T2 weighting on the diffusivity calculation. However, tissue components with a shorter T2 value will contribute less to the measured signal (and therefore the calculated diffusion tensor) than components with longer T2 values, particularly in the M2-SE acquisition. The exact influence of T2 on measured diffusion will depend convolutedly on T2 values within the multiple tissue compartments and the trans-membrane water exchange rates between them. A similar multi-compartment problem also applies in regards to differences in the T1 weighting of the two sequences.

Another factor contributing to the differences in results is the differing sensitivity to cardiac motion of the two sequences. The STEAM sequence uses short diffusion encoding gradients, which minimises the detrimental effects of motion during the gradients (although inter-gradient motion is problematic). In contrast, the spin-echo sequence requires a much larger gradient area and the gradients are further extended in time in the M2-SE sequence in order to compensate for velocity and acceleration. While the M2 encoding gradients have a reduced sensitivity to motion, it requires that higher order motion components are minimal while they are run.

The higher failure rate of the M2-SE sequence at the sweet-spot and in diastasis could be attributed to uncompensated cardiac motion during the long diffusion encoding gradients, which is perhaps also consistent with the primary eigenvalues substantially exceeding that of free water in some pixels when using this sequence (Fig. 8). The largest number of studies failed in diastole (7/15) and the diastolic M2-SE image quality score significantly increased with lengthening RR-intervals. The magnitude of cardiac motion is low in diastasis, but the motion trajectories may be complex. Welsh et al. [18] considered the effects of velocity, acceleration and jerk compensation on diffusion encoding gradients and concluded that 2nd order compensation was sufficient in rats. However, the increasing gradient duration as the motion compensation order increases, also makes it increasingly likely that more complex motion will occur during them. Recent work demonstrated increased cardiac motion and eddy current related signal loss in M2-SE sequences than in STEAM sequences [43], although very little of the difference in MD between sequences could be attributed to this. High performance gradient systems, such as those now commercially available with maximum gradient strength of 80mT⋅m^−1^ at slew rates of 200T⋅ [m⋅s]^−1^ may partially address the residual motion sensitivity of M2-SE. Stoeck et al. [20] used a maximum gradient amplitude/slew rate of 80mT⋅m^−1^/100T⋅[m⋅s]^−1^ to reduce the total M2 diffusion encoding time to 50 ms, versus 62 ms achieved here (TE = 73 ms versus TE = 76 ms here). In a study of the sensitivity of the M2-SE technique to gradient strength, 2/6 systolic acquisitions failed when maximum gradient strengths were only 30mT⋅m^−1^ [42]. In order to fully understand the effects of cardiac motion on the results and success rates of both sequences in various points throughout the cardiac cycle, future work should consider computational simulations, extending the work of Mekkaoui et al. [44]. One of the difficulties with such simulations is that they will require in-vivo measures of 3D motion trajectories covering the whole cardiac cycle, accurate to at least 3rd order motion (jerk). Myocardial phase velocity mapping, with 3-direction encoding and retrospective cardiac gating [45] may be suited to acquiring such data.

Investigations of the optimum timing of the M2-SE sequence [20, 46] have found that triggering between 15% and 77% of the time from the R-wave to peak-systole (during systolic contraction) resulted in an acceptable level of motion related signal loss (although time points beyond peak-systole were not analysed). In this work, we found the highest success rate when the centre of k-space was timed to peak systole, equivalent to triggering at 75% of peak systole (TE = 75 ms, time to peak systole 300 ms). In comparison to other work, we found lower success rates at the systolic sweet-spot, which we defined as a constant 150 ms from the R-wave based on previous work considering the predicted effects of strain on STEAM data [27]. However, the three M2-SE acquisitions which failed at the sweet-spot correspond to subjects with the maximum (100 bpm) and minimum (50 and 55 bpm) mean heart rates, suggesting that a fixed trigger time may not be best suited for M2-SE imaging.

A further difficulty with acquiring M2-SE data in diastasis is that the longer acquisition window frequently caused the scanner to miss the next R-wave. Based on DICOM timestamp information we estimate the proportion of missed triggers as 7% for diastolic M2-SE and 0.1–0.6% in other acquisitions. These missed triggers extend breath-hold durations and perturb the longitudinal magnetisation’s steady-state, which is likely to result in errors in the calculated diffusion tensor. In earlier pilot work [24], we achieved a higher success rate for M2-SE imaging in diastasis (15/20 acquisitions successful) by triggering M2-SE to alternate R-waves (TR = 2RR-intervals). Principal component analysis – temporal maximum intensity projection (PCA-TMIP) post-processing technique [47] and related methods [48] could potentially be combined with M2-SE sequences to palliate its motion sensitivity. Alternatively, pharmacological methods of increasing the length of diastasis may be useful [49].

The strain history of the imaged tissue during the diffusion time affects the measured diffusion [12] and the long diffusion time of STEAM means it is more affected than M2-SE. We found significant correlations between peak radial strain and the difference between systolic and diastolic FA and E2A using STEAM (*p* < 0.05). We did not perform a full strain correction [10, 50]. Currently the only model [12] describing the modification of the diffusion tensor in response to strain assumes that the myocardium can be modelled as a homogeneous elastic medium. While it is clear that diffusion measured with a monopolar STEAM sequence over 2 cardiac cycles should be affected by cyclical strain, the complex multi-scale structure of the heart means that the exact effects of strain are more complex than those described by the existing model. We have recently highlighted issues with the results provided by the model by comparing ex-vivo and in-vivo STEAM DT-CMR in pigs [13]. The correlation between E2A and peak radial strain could be due to the strain effect, but E2A mobility (systole-diastole) obtained using the strain insensitive M2-SE technique also correlates with peak radial strain (*p* = 0.01). Furthermore, it is logical to expect a correlation between E2A mobility and radial strain even in the absence of the effects of strain on the DT-CMR acquisition. The change in sheetlet angle represents the primary mechanism behind wall thickening [1] and we have recently validated these changes in a pre-clinical model [6]. However, we do expect some contribution of strain to the E2A mobility measured with STEAM and this may be reflected in the correlation (*p* < 0.01) of differences in E2A between sequences with strain in both diastole and systole. Recent work comparing arrested and beating heart measures of E2A in relaxed and contracted states, estimated the maximum component of E2A mobility attributable to strain [6] as ~17%. The median E2A mobility measured in this study using STEAM of 40^o^ compared to 11^o^ measured with M2-SE (Supporting Table S1) suggests that strain is not the only contributor to this difference. A component of the difference between parameters other than E2A may also be the result of strain effects. While an ex-vivo study has demonstrated changes in eigenvalues and FA between contracted and relaxed states, MD was maintained [51]. As a result, we can estimate the maximum possible contribution of strain to MD measured with STEAM as the difference between the sweet-spot and the diastolic or systolic values. These differences of 0.05 × 10^−3^mm^2^⋅s^−1^ and 0.06 × 10^−3^mm^2^⋅s^−1^ (calculated from values in Supporting Table S1) at systole and diastole respectively, suggest that strain is not a major contributor to the much larger difference in MD observed between the two sequences. Indeed, even at the sweet-spot, where the existing model predicts minimal strain effects for STEAM data, there are large differences between the results from the two sequences.

**Fig. 11:**
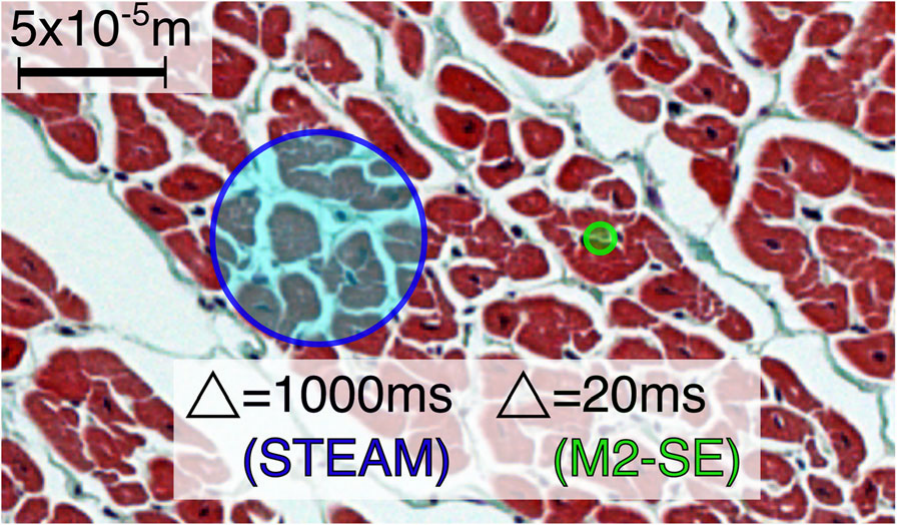
Distances diffused during the diffusion time. Comparison of the average distance diffused by water molecules during the diffusion time of the STEAM (blue circle) and M2-SE (green circle) sequences. The distances are super-imposed on a histology image cut from a pig heart perpendicular to the myocyte long axis. Based on a diffusivity of 3 × 10^−3^mm^2^s^−1^, free water molecules at 37 °C would diffuse around 75 μm during the diffusion time of the STEAM sequence, but only 10 μm during the diffusion time of the M2-SE sequence

The difference in diffusion time between these two sequences is expected to result in changes in the DT-CMR results [21, 52]. During the diffusion time of the STEAM sequence (~1 cardiac cycle, ~1000 ms), free water molecules would diffuse a root mean square distance of around 75μm (D = 3 × 10^−3^mm^2^⋅s^−1^ at 37 °C (41)), while the equivalent distance for the M2-SE sequence (diffusion time ~20 ms) is only around 10μm, which is less than typical cardiomyocyte dimensions (see Fig. 11). This limits the number of diffusing water molecules interacting with cellular structures and, while meaningful theoretical predictions of the strength of this effect are impossible without complex numerical simulations, previous work in ex-vivo tissue can provide some insights. Kim et al. [52] studied the diffusion time dependence in fresh samples of calf heart. Increasing the diffusion time from 33 ms to 412 ms resulted in an increase of FA from 0.40 ± 0.07 to 0.59 ± 0.06 and a reduction in MD from 1.59 ± 0.12 × 10^−3^mm^2^⋅s^−1^ to 1.33 ± 0.08 × 10^−3^mm^2^⋅s^−1^. Despite the obvious limitations of comparing with ex-vivo tissue, these results suggest that the changes we observe here between sequences are predominantly a consequence of diffusion time effects. A recent review by Kiselev [53] suggests that probing cellular muscle structure is only possible with the longer diffusion times available with stimulated echoes and we might expect reductions in the uncertainty of angular DT-CMR measures with such sequences.

A further consequence of using a full cardiac cycle as the diffusion time in the STEAM sequence is that the b-value is modulated by variations in the RR-interval. In order to account for this b-value variation we corrected the b-values used in the tensor reconstruction based on the time stamps of the DICOM images. However, the time dependence of the measured diffusion is not accounted for in this correction, but should be small for typical variations in the RR-interval (the mean of the intra-subject standard deviation of the RR-interval was 5 ms) [52].

Using phantom data we demonstrate a 1.75 factor increase in SNR per image using the M2-SE sequence over STEAM. Due to the longer T1 and shorter T2 of myocardial tissue, the theoretical SNR ratio is lower in vivo than in the phantom. Based on Eq. 1 the theoretical SNR ratio in vivo is SNR_SE_/SNR_STEAM_ = 1.37, assuming myocardial T2 = 45 ± 4 ms (T2-prepared method [54], measured in 20 subjects in earlier work [24]), T1 = 1327 ± 59 ms (modified Look Locker 5(3)3 [39, 40], in the same 20 subjects) and an RR interval of 970 ms. In the “b0” data we measured a median [interquartile range] in-vivo SNR ratio of SNR_SE_/SNR_STEAM_ 0.78 [0.38], 0.68 [0.23] and 0.59 [0.38] at systole, sweet-spot and diastole. These ratios are lower than the results of von Deuster et al. [21], who measured SNR efficiency and less than theoretically predicted. SNR per image measured using the multiple repetitions method is affected not only by true image noise but also by variations in signal intensity due to motion related signal loss, imperfect registration and residual blood signal. In this work, EPI readouts, receive gain, receive coils, reconstruction schemes and image scaling were matched between sequences and it is therefore reasonable to assume that image noise is similar (and we have confirmed this is so in phantom experiments, not included here). Therefore, we use the ratio between mean signal intensities in the two sequences as a surrogate for SNR ratio with fewer confounding effects than the multiple repetitions method. The median [interquartile range] signal ratios (M2-SE/STEAM) were 1.01[0.21], 0.96[0.12] and 1.19[0.34] at systole, sweet-spot and diastole; suggesting that a substantial fraction of the reduction in M2-SE SNR per image measured using the multiple repetitions method relative to theory is due to the confounding factors discussed above. The remaining difference between mean signal ratio and the theoretical SNR ratio could be a consequence of sequence differences in the in-plane excitation profiles. Small M2 gradients are used as crushers around the 180^o^ pulse in the “b0” acquisition and higher order motion during these gradients may also contribute to the reduction in measured mean signal ratio relative to theory. In practice the SNR per unit acquisition time (as calculated by von Deuster et al. (21)) of the M2-SE sequence is boosted beyond that measured here by the additional averages that can be acquired due to its shorter TR compared to STEAM. However, STEAM allows much higher b-values to be used with little penalty in TE [33] and therefore a potential increase in diffusion contrast to noise relative to the M2-SE sequence.

Our more comprehensive findings differ from the smaller studies of both von Deuster et al. [21] and Stoeck et al. [20], who did not report any unsuccessful DT-CMR acquisitions. At the sweet-spot we found similar mean FA using both sequences and MD using STEAM, but higher MD values using M2-SE (1.69 ± 0.15 × 10^−3^mm^2^⋅s^−1^ vs. 1.43 ± 0.06 × 10^−3^mm^2^⋅s^−1^ (21)). There were a number of key differences between our work and these previous studies. While we imaged at 3 T with a widely available gradient strength Stoeck et al. [20] and von Deuster et al. [21] used a high strength gradient system (80mT⋅m^−1^, 100 T⋅[m⋅s]^−1^) at 1.5 T. We used parallel imaging to shorten the EPI readout and used optimal diffusion encoding directions for gradient performance to achieve a similar TE for M2-SE (76 ms vs. 73 ms (20) vs. 70 ms [21]) and a shorter STEAM TE (25 ms vs. 31 ms(21)). We imaged at three cardiac phases rather than only at the systolic sweet spot. We acquired data during multiple breath holds, as did von Deuster et al. [21], which we have found to be a more reliable method of acquiring STEAM data, although M2-SE acquisitions may be well suited to navigator gated free-breathing acquisitions [20, 21]. There is also the potential confounding issue of experience. While our group has ~6 years experience acquiring and processing STEAM DT-CMR data and one experienced operator (ADS: 11 years CMR experience, 5 years DT-CMR experience, >40 subjects imaged with M2-SE) imaged all 15 subjects, M2-SE is a relatively new and evolving technique [55]. Therefore, we biased our study to the advantage of M2-SE acquisitions by selecting trigger times for all systolic and diastolic acquisitions based on the optimal timing for M2-SE acquisitions.

## Conclusions

Using a clinical 3 T scanner with standard gradients, both DT-CMR sequences perform equally well at the systolic pause and are successful in the majority of subjects. STEAM can also be used reliably in diastole, but 2nd order motion compensation of M2-SE did not appear to adequately cope with the low magnitude but complex trajectory of the motion at this cardiac phase. There were clear differences between the results obtained using each of these methods and only a few of these differences correlated with measures of strain. These differences are predominantly the result of the 2 orders of magnitude difference in diffusion time between these two techniques. While M2-SE may be more suited to free-breathing studies or imaging subjects with arrhythmia, STEAM is a more robust acquisition when diastolic imaging is required. It is, therefore, vital to understand the differences between these two methods and obtain normal values for the sequence of choice when planning future patient studies.

## Additional file

Additional file 1: Figures S1–S6 and **Tables S1** and **S2.** (PDF 3207 kb)

## Abbreviations

B: Diffusion weighting
b_main_: Main diffusion weighting
b_ref_: Reference diffusion weighting
DENSE: Displacement encoding with stimulated echoes
DT-CMR: Diffusion tensor cardiovascular magnetic resonance
E1, E2, E3: The first, second and third eigenvalues respectively
E2A: Absolute value of the second eigenvector angle
EPI: Echo planar imaging
FA: Fractional anisotropy
HA R^2^: Pearson correlation coefficient of the linear regression of the helix angle with transmural depth
HA RMSE: Root mean square error of the linear regression of the helix angle with transmural depth
HA: Helix angle
HAG: Helical angle gradient
LV: Left ventricle/left ventricular
M2-SE: First and second order motion compensated spin-echo
MD: Mean diffusivity
PCA-TMIP: Principle component analysis – temporal maximum intensity projection
RF: Radiofrequency
ROI: Region-of-interest
SE: Spin echo
SENSE: Sensitivity encoding
SNR: Signal-to-noise ratio
STEAM: Stimulated echo acquisition mode
TA std.: Standard deviation of the transverse angle
TA: Transverse angle
TE: Echo time
TR: Repetition time
