# Supplementary material for: An in-vivo comparison of stimulated-echo and motion compensated spin-echo sequences for 3 T diffusion tensor cardiovascular magnetic resonance at multiple cardiac phases
Source: J Cardiovasc Magn Reson. 2018 Jan 3;20:1. doi: 10.1186/s12968-017-0425-8 (PMC5753538; doi:10.1186/s12968-017-0425-8)
Supplement: Additional file 1: Figures S1–S6 — and Tables S1 and S2. (PDF 3207 kb) [file 12968_2017_425_MOESM1_ESM.pdf]

Supporting Figure S1: Example raw STEAM images corresponding to the DT-CMR maps shown in figure 4. Images rejected based on artefact and motion related signal drop out are highlighted in red.

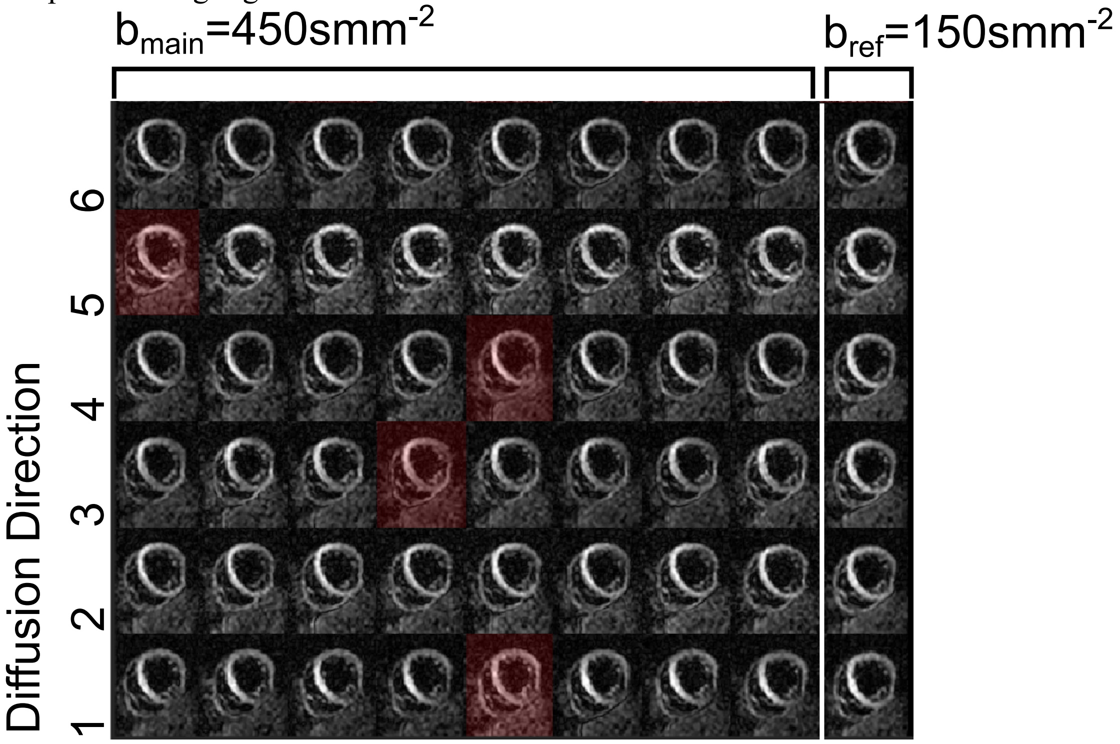

Supporting Figure S2: Example raw M2-SE images corresponding to the DT-CMR maps shown in figure 4. Images rejected based on artefact and motion related signal drop out are highlighted in red.

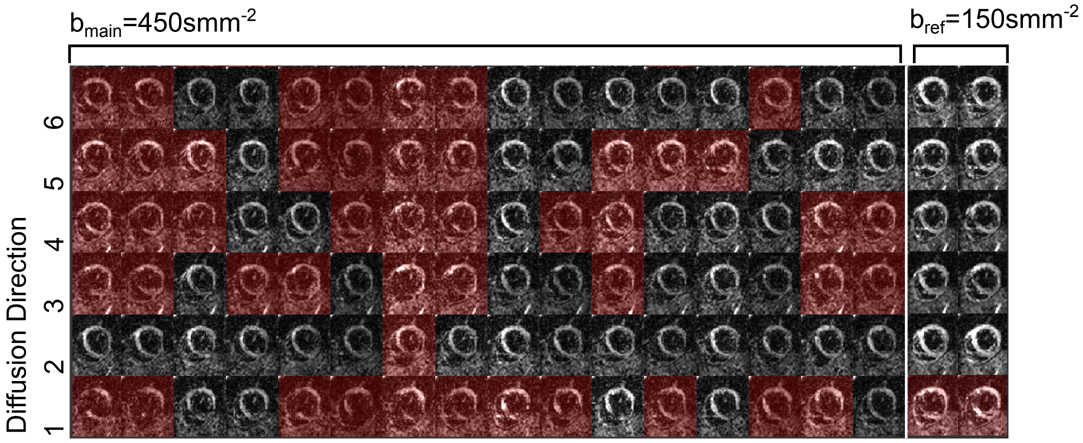

Supporting Figure S3: Plots of the subject-wise mean RR-interval with subjective image score for acquisitions where the Spearman correlation was significant ($p<0.05$). Spearman's Rho and the corresponding p-value are shown on the plots.

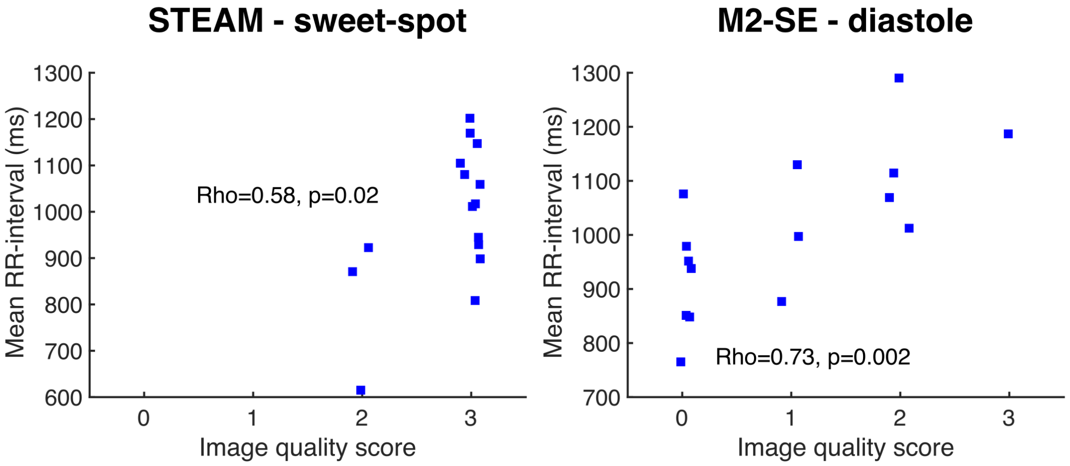

Supporting Figure S4: A comparison of the mean left ventricular tensor mode obtained from both sequences using the cDTI data at all three time points in the cardiac cycle. The points are colour coded by subject. Median and interquartile ranges are shown in black (STEAM) and gray (M2-SE) with the p-value obtained from a Wilcoxon signed rank test comparing the sequences at each time point shown above the data where p<0.05.

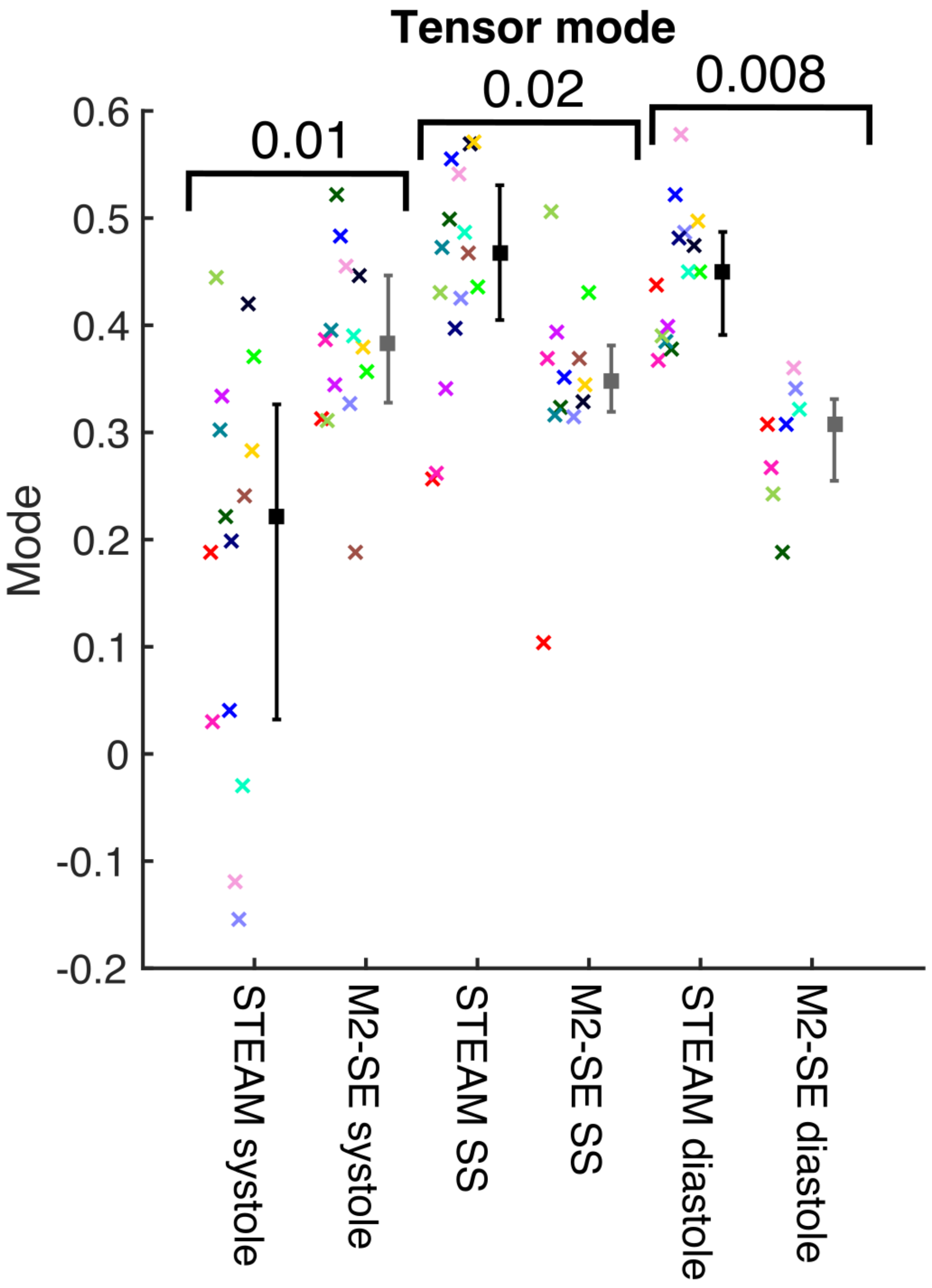

Supporting Figure S5: A comparison of the mean left ventricular HA R^2 (a), HA root mean square error (b), transverse angle standard deviation (c), obtained from both sequences using the DT-CMR data at all three time points in the cardiac cycle. The points are colour coded by subject. Median and interquartile ranges are shown in black (STEAM) and gray (M2-SE) with the p-value obtained from a Wilcoxon signed rank test comparing the sequences at each time point shown above the data where p<0.05.

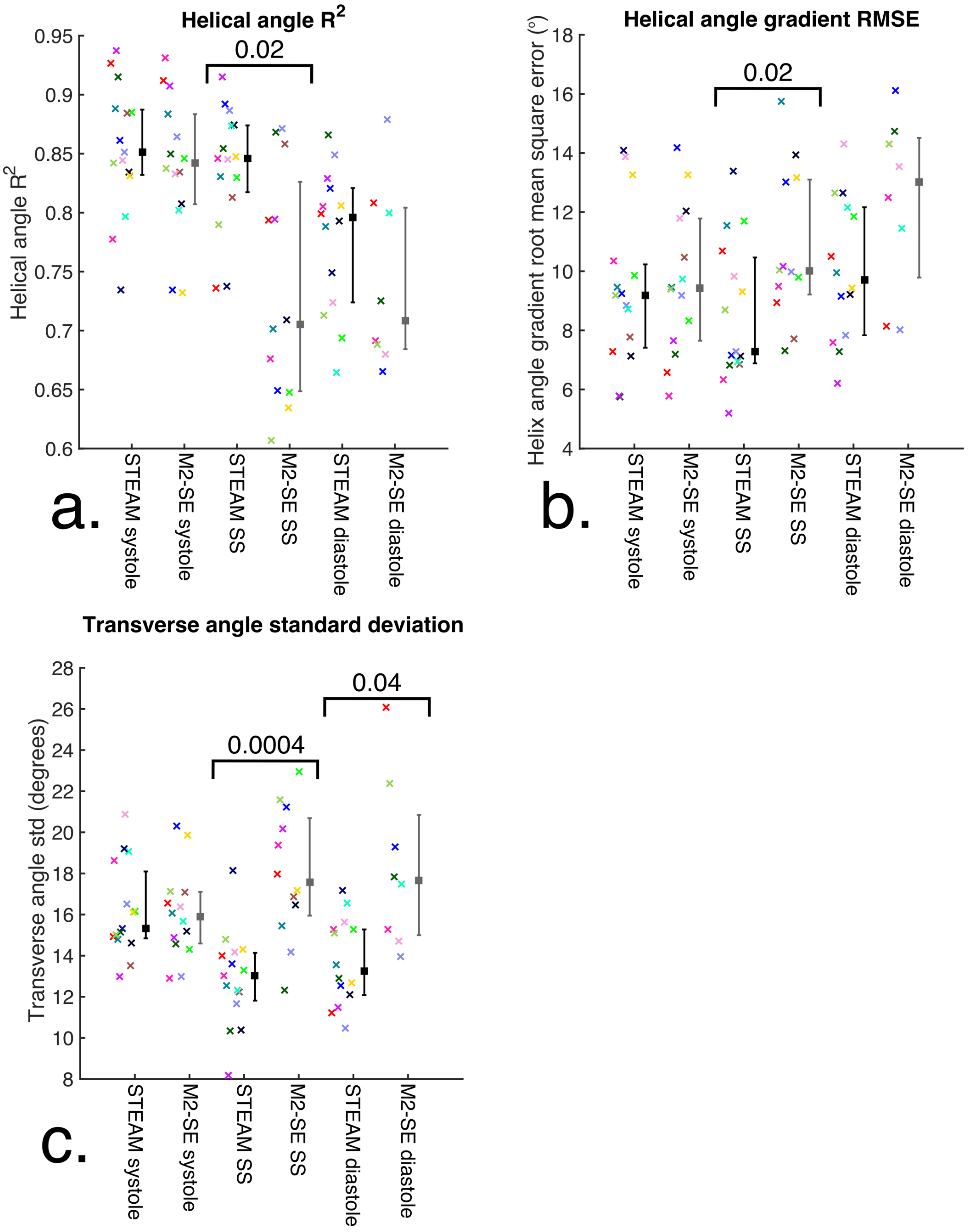

Supporting Figure S6: A comparison of the SNR measured in a mesocardial septal region of interest (b_{ref}=30/34 smm^{-2} images) (e) and the mean signal in the same region of interest (f) obtained from both sequences using the DT-CMR data at all three time points in the cardiac cycle. The points are colour coded by subject. Median and interquartile ranges are shown in black (STEAM) and gray (M2-SE) with the p-value obtained from a Wilcoxon signed rank test comparing the sequences at each time point shown above the data where p<0.05.

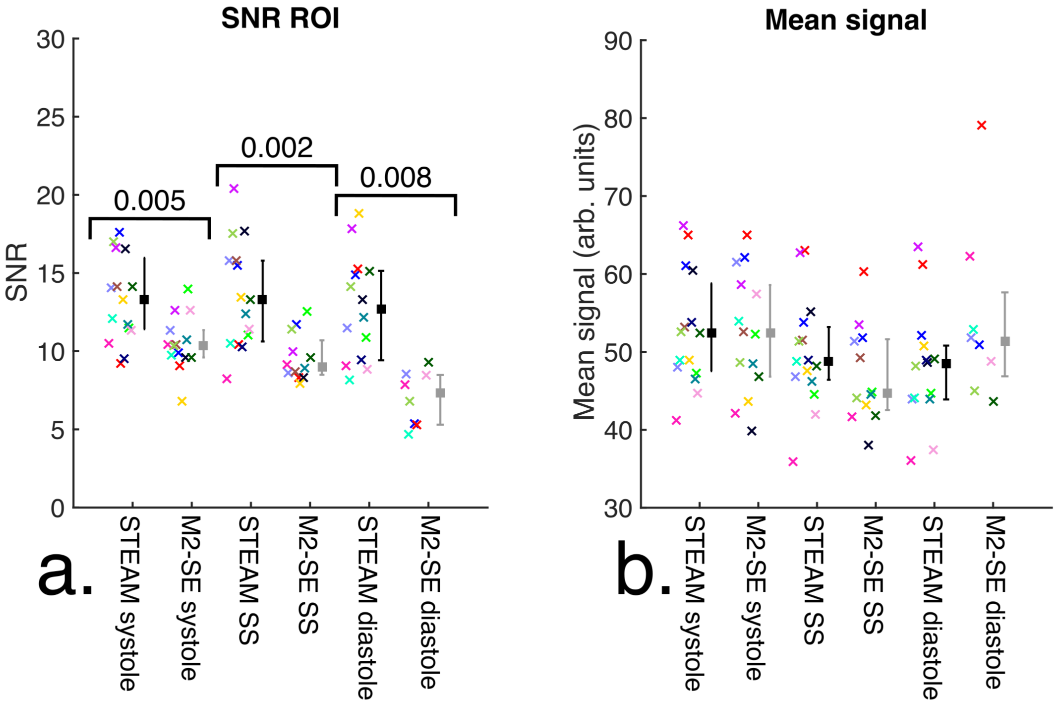

Supporting Table S1: A comparison of DT-CMR results between cardiac phases using both STEAM and M2-SE.

	STEAM				M2-SE			
Parameter	Systole	SS	Diastole	p	Systole	SS	Diastole	p
MD $\times 10^{-3}$ mm^2/s	1.02† (0.14)	1.07 (0.12)	1.13§ (0.08)	0.02	1.46 (0.43)	1.70 (0.18)	1.78 (0.34)	0.2
FA	0.47*† (0.03)	0.57§ (0.05)	0.61§ (0.04)	10^{-6}	0.40 (0.09)	0.45 (0.08)	0.41 (0.07)	0.5
E2A [median] °	55*† (10)	24§† (16)	15*§ (4)	10^{-7}	37 (9)	26 (10)	26 (5)	0.1
E2A [mean] °	52*† (9)	28§† (14)	20*§ (6)	10^{-6}	39 (7)	31 (11)	30 (5)	0.1
HAG °/%	-0.72† (0.14)	-0.68† (0.18)	-0.53*§ (0.26)	0.03	-0.77 (09)	-0.46 (0.39)	-0.45 (0.19)	0.1
Mode	0.21*† (0.30)	0.45§ (0.14)	0.45§ (0.10)	10^{-4}	0.36 (0.17)	0.34 (0.05)	0.29 (0.07)	0.1
E1 $\times 10^{-3}$ mm^2/s	1.52*† (0.25)	1.82§† (0.15)	2.02*§ (0.17)	10^{-4}	2.08 (0.43)	2.60 (0.38)	2.59 (0.30)	0.1
E2 $\times 10^{-3}$ mm^2/s	1.01 (0.14)	0.94 (0.14)	0.97 (0.10)	0.3	1.34 (0.43)	1.54 (0.14)	1.67 (0.31)	0.2
E3 $\times 10^{-3}$ mm^2/s	0.56*† (0.15)	0.50§ (0.13)	0.45§ (0.12)	0.01	0.94 (0.41)	0.97 (0.15)	1.07 (0.41)	0.1
TA std °	15.7*† (3.7)	13.2§ (2.5)	13.2§ (3.2)	10^{-5}	15.6 (4.1)	18.9 (7.1)	18.6 (7.1)	0.1
HA R^2	0.85† (0.06)	0.85† (0.04)	0.80*§ (0.10)	0.01	0.86 (0.07)	0.73 (0.22)	0.71 (0.12)	0.2
HA RMSE °	9.2 (3.1)	8.0 (3.7)	9.7 (4.3)	0.05	8.2 (2.8)	9.7 (1.1)	13.4 (6.6)	0.1
SNR	12.6 (5.2)	12.8 (5.3)	12.7 (5.7)	0.2	10.12† (0.82)	9.4† (2.8)	7.3*§ (3.2)	0.01
Mean signal	51*† (13)	48.5§ (7.5)	48.4§ (6.9)	10^{-4}	55 (15)	47 (10)	51 (17)	0.03

Values are quoted as median (interquartile range: 75th – 25th centile) and statistics were calculated with N=14 for STEAM and N=6 for M2-SE due to the rejected datasets. While E2A [median] is used throughout this paper, E2A [mean] was used in previous work and is included here for comparison.

§ - p<0.017 vs. systole, * - p<0.017 vs. sweet spot, † - p<0.017 vs. diastole. p=0.17 is equivalent to p=0.05 after Bonferroni correction for 3 comparisons.

Supporting Table S2: Correlations between cardiac phases and between sequences with peak radial and circumferential strains.

Sequence and time point	Strain parameter	DT-CMR result	Normalised (beta) coefficient from linear regression	R^2	p
STEAM (systole – diastole)	Peak radial	FA	-0.54	0.29	0.05
STEAM (systole – diastole)	Peak radial	E2A	0.73	0.53	0.003
M2-SE (systole – diastole)	Peak radial	E2A	0.83	0.69	0.01
Systole (STEAM – M2-SE)	Peak radial	E2A	0.70	0.49	0.005
Diastole (STEAM – M2-SE)	Peak radial	E2A	0.89	0.79	0.002

E2A is measured in °.
